# Correction to: Novel interaction between neurotrophic factor-α1/carboxypeptidase E and serotonin receptor, 5-HTR1E, protects human neurons against oxidative/neuroexcitotoxic stress via β-arrestin/ERK signaling

**DOI:** 10.1007/s00018-023-04711-0

**Published:** 2023-02-20

**Authors:** Vinay Kumar Sharma, Xuyu Yang, Soo-Kyung Kim, Amirhossein Mafi, Daniel Saiz-Sanchez, Patricia Villanueva-Anguita, Lan Xiao, Asuka Inoue, William A. Goddard, Y. Peng Loh, Leila Toulabi

**Affiliations:** 1 Section on Cellular Neurobiology, Eunice Kennedy Shriver National Institute of Child Health and Human Development, National Institutes of Health, 49, Convent Drive, Bldg 49, Rm 6A-10, Bethesda, MD 20892 USA; 2 Materials and Process Simulation Center, California Institute of Technology, Pasedena, CA 91125 USA; 3 Neuroplasticity and Neurodegeneration Laboratory, Medical School, Regional Center for Biomedical Research, University of Castilla-La Mancha, 13071 Ciudad Real, Spain; 4 Graduate School of Pharmaceutical Sciences, Tohoku University, Sendai, Miyagi 980-8578 Japan

**Correction: Cellular and Molecular Life Sciences (2021) 79:24** 10.1007/s00018-021-04021-3

In the published article, the author name Leila Toulabi was missed during the proof stage and it has been now updated.

Section on Cellular Neurobiology, Eunice Kennedy Shriver, National Institute of Child Health and Human Development, National Institutes of Health, 49, Convent Drive, Bldg 49, Rm 6A‑10, Bethesda, MD 20892, USA.

The original article has been updated.

